# Skeletal muscle atrophy is attenuated in tumor-bearing mice under chemotherapy by treatment with fish oil and selenium

**DOI:** 10.18632/oncotarget.3483

**Published:** 2015-03-08

**Authors:** Hang Wang, Tsung-Lin Li, Simon Hsia, I-Li Su, Yi-Lin Chan, Chang-Jer Wu

**Affiliations:** ^1^ Department of Food Science, National Taiwan Ocean University, Keelung, Taiwan; ^2^ Genomics Research Center, Academia Sinica, Taipei, Taiwan; ^3^ Institute of Biomedical Nutrition, Hung Kuang University, Taichung, Taiwan; ^4^ Antai Medical Care Cooperation Antai Tian-Sheng Memorial Hospital, Pingtung, Taiwan; ^5^ Department of Life Science, Chinese Culture University, Taipei, Taiwan; ^6^ Center of Excellence for the Oceans, National Taiwan Ocean University, Keelung, Taiwan

**Keywords:** muscle atrophy, cachexia, fish oil, selenium, chemotherapy

## Abstract

Chemotherapy can cause cachexia, which is manifested by weight loss, inflammation and muscle atrophy. However, the mechanisms of tumor and chemotherapy on skeletal muscle proteolysis, remained unclear. In this report, we demonstrated that tumor-induced myostatin in turn induced TNF-α, thus activating calcium-dependent and proteasomal protein degradation. Chemotherapy activated myostatin-mediated proteolysis and muscle atrophy by elevating IL-6. In tumor-bearing mice under chemotherapy, supplementation with fish oil and selenium prevented a rise in IL-6, TNF-α and myostatin and muscle atrophy. The findings presented here allow us to better understand the molecular basis of cancer cachexia and potentiate nutrition supplementation in future cancer chemotherapy.

## INTRODUCTION

Cancer cachexia influences >80% advanced cancer patients and accounts for 30% cancer-related deaths [1]. Cancer cachexia is defined as >5% weight loss, which is closely associated with muscle weakness, fatigue, anorexia, low lean body mass and certain abnormal physiological symptoms, such as inflammation, anemia, low serum albumin. The loss of 5%, 10% or 15% of the total body mass is further graded as mild, moderate or severe cachexia, respectively. Both the weight loss and the rate of weight loss are well correlated to cancer mortality [2]. Apart from the consequences of cancer *per se*, chemotherapy also provokes severe anorexia and weight loss [3]. For example, docetaxel (Taxotere), a semisynthetic taxane, is used to treat breast, prostate, and non-small cell lung cancers (NSCLC) [4], while it otherwise gives rise to serious cachexia.

The loss of muscle masses is a hallmark of cancer cachexia, which is manifested with degradation of myofibrillar proteins [5] and often come with decrease of protein synthesis [6]. There are three major proteolytic pathways in skeletal muscle catabolism: 1. The lysosomal system (e.g. the cysteine protease cathepsin L (CaspL)) that degrades extracellular proteins and cell receptors [7]. 2. The calcium-activated system (e.g. calpains I and II) that is involved in tissue injury, necrosis, and autolysis [8]. 3. The ubiquitin-proteasome pathway that functions along with the calpain system for disassembly/degradation of muscle myofilaments [9]. The cancer cachexia-induced muscle loss is multifactorial making cancer therapy intricate. Cytokines greatly influence development of cachexia, in which proinflammatory cytokines TNF-α and IL-6 play a much greater role. In the ubiquitin-proteasome proteolytic pathway TNF-α initiates muscle atrophy through up-regulating cathepsins (B and L) and ubiquitins [10]. IκBs however regulate the proteasomal pathway [11, 12]. Myostatin, an extracellular protein, negatively regulates muscle masses [13] by inhibiting myoblast proliferation [14]. The fact is that myostatin exerts its effect through down-regulating muscle regulatory factors MyoD and myogenin [15]. Myostatin then induces MAFbx/atrogin-1 and the proteasomal activity [16]. While much have been learned, the interplay between lung cancer and/or chemotherapeutics that bring about muscle atrophy remain far from clear.

There is so far no effective way to counteract cancer-provoked muscle wasting, while some recent findings may provide practical solutions. For example, we reported that nutritional supplementation with a combination of fish oil [17] and selenium yeast (se) can lessen body weight loss by enhancing anti-tumor immunity [18]. Fish oil was also reported able to relieve muscle fatigue [19], thus improving physical soundness [20]. The skeletal muscle depletion may partially be ascribed to reduction of plasma n-3 fatty acids in NSCLC patients [21, 22]. Selenium (Se) was recently reported to be a chemopreventive agent [23], which may prevent mammary epithelial cells from oxidative DNA damage [24] in addition to promoting cancer cell apoptosis [25].

To address above concerns we first elucidated the formation of cancer chemotherapy-induced cachexia through three cachectic models. Our results determined that TNF-α facilitates development of muscle atrophy and that IL-6 acts on the onset of proteolysis during chemotherapy. We concluded that fish oil and selenium are beneficial to tumor chemotherapy-induced cachexia.

## RESULTS

### Cachexia parameters in tumor-bearing mice

**Figure 1:**
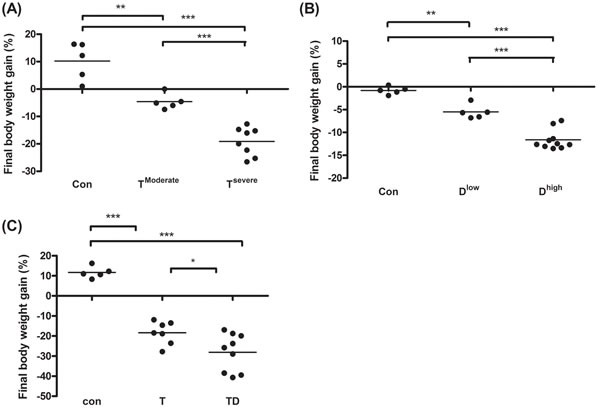
Final body weight gain for line-1, docetaxel or combined treatment mice After sacrifice we scaled mice body weight into two groups according to the body weight loss percentage. The body weight loss >10% or <10% is designated as severe cachexia or moderate cachexia. (A and C) On day 35 and 42 after tumor implantation, we harvest tumor and subtract tumor weight from final body weight to calculate the percentage of body weight loss for line-1 (A), docetaxel (B), or combined chemotherapy (C) treatment mice, respectively. Data show mean ± SD, n = 5–10 mice/group, each value is an average of three independent experiments. *p < 0.05, **p < 0.01, and ***p < 0.001 denote levels of significant differences between groups.

In animal study, mice were divided into two groups, severe and moderate cachexia as described previously (protocol #1) [18]. The former is redefined as >10% body weight loss for simplicity. In Table 1A, the carcass weight of the tumor-bearing mice (TB) is lighter than that of control mice [26]. In terms of tumor weight, the severe cachectic mice are 2-fold heavier than the moderate (Table 1A). The body weight, however, gained about 10.2% in normal mice, as opposed to the body weight losses, 5.6% and 19.1%, in moderately and severely cachectic mice, respectively (Figure 1A). The body weight loss can thereby index the severity of the tumor-induced cachexia. The loss of muscle/fat (e.g., epididymal fat) accounts for the major weight loss (Table 1A). Though anorexia usually comes with cancer cachexia, food intake made no difference between the experiment and control groups (Table 1B). Given that inflammation and hypoalbuminemia are two diagnostic indices for cachexia [27], the level of albumin in the severe cachectic mice was not surprised significantly lower (*p* < 0.05, T^Moderate^ vs. T^Severe^) than that in the moderate (Table 2A). Serum TNF-α and IL-6 in the severe cancer group were higher than those in control, whereas serum IL-1β leveled off in all groups (Table 2A).

**Table 1:** Cachexia-induced organ mass changes

(A) Body, tumor and organ weights (protocol #1)						
Treatment	N	BW(g)	TW (g)	CW (g)	eWAT (mg)	mG (mg)
Con	5	28.8±1.9		28.8±1.9	362.8±71.8	180.0±36.7
T^Moderate^	5	28.0±2.1	4.1±1.4	23.9±1.2***	275.7±53.8*	150.3±21.9
T^Severe^	8	30.8±3.3	8.3±3.1^†^	22.5±2.8***	116.7±50.5***,^†^	134.2±29.1**

(B) Body and organ weights (protocol #2)						
Treatment	N	IW(g)		CW (g)	eWAT (mg)	mG (mg)
Con	5	26.0±0.5		25.8±0.6	235.5±67.1	170.0±22.7
D^low^	5	26.2±0.8		24.8±0.9*	185.1±36.2*	140.3±19.4*
D^high^	10	25.4±0.5		22.5±0.5**	190.2±20.5*	103.2±23.8**,^†^

(C) Body, tumor and organ weights (protocol #3)						
Treatment	N	BW(g)	TW (g)	CW (g)	eWAT (mg)	mG (mg)
Con	5	30.8±0.8		30.8±0.8	377.2±63.4	236.2±29.8
T	7	29.6±2.4	8.8±0.7	20.8±1.3*	178.1±44.2***	182.3±31.4*
TD	9	24.7±3.3**	5.7±2.3^†^	19.0±0.7**	162.9±55.5***	123.1±32.3***

BW = Body weight, IW =Initial body weight, TW = Tumor weight, CW = Carcass weight, eWAT = epididymal White Adipose Tissue, mG = muscle Gastrocnemius. Data represent mean ± SD. Statistical significance was determined by one-way ANOVA.

* *p* < 0.05

** *p* < 0.01

*** *p* < 0.001 presents significant differences from Con mice.

† *p* < 0.05 presents significant differences from Cancer^Moderate^ or D^low^ or T mice.

**Table 2:** Cachectic parameters

(A) Cachectic parameters (protocol #1)						
Treatment	N	Albumin (g/dl)	TNF-α (pg/ml)	IL-6 (pg/ml)	IL-1β (pg/ml)	FI
Con	5	2.90±0.09	30.6±4.3	14.0±3.4	18.5±6.4	5.2±1.4
T^Moderate^	5	2.20±0.05***	69.4±11.7	19.4±5.2	23.4±6.2	5.4±2.1
T^Severe^	8	1.98±0.03***,^†^	98.8±19.2**	27.8±4.8**	28.2±8.0	4.8±2.5

(B) Cachectic parameters (protocol #2)						
Treatment	N	Albumin (g/dl)	TNF-α (pg/ml)	IL-6 (pg/ml)	IL-1β (pg/ml)	FI
Con	5	2.96±0.08	36.9±6.8	13.4±3.8	15.7±5.2	5.5±1.4
D^low^	5	2.70±0.04**	40.1±7.4	28.6±7.2	33.2±8.1*	4.4±1.1*
D^high^	10	2.49±0.02***,^†^	40.9±4.4	47.3±6.2**	58.6±7.0**	2.9±2.5**

(C) Cachectic parameters (protocol #3)						
Treatment	N	Albumin (g/dl)	TNF-α (pg/ml)	IL-6 (pg/ml)	IL-1β (pg/ml)	FI
Con	5	2.98±0.11	33.8±3.3	14.0±3.4	18.1±5.0	6.4±2.5
T	7	2.16±0.07*	78.4±9.4**	21.2±4.2	26.7±6.2	6.5±3.1
TD	9	2.04±0.05*	66.3±7.9*	25.7±3.8*	30.2±6.4	6.5±2.8

FI = Daily food intake (g/mice/day). Data represent mean ± SD. Statistical significance was determined by one-way ANOVA.

* *p* < 0.05

** *p* < 0.01

*** *p* < 0.001 presents significant differences from Con mice.

† *p* < 0.05 presents significant differences from Cancer^Moderate^ or D^low^ or T mice.

### Characteristics of chemotherapy-induced cachexia

In Table 1B, the basic indexes, carcass weight, food intake and serum albumin, significantly decline in the docetaxel-treated mice. These indexes dropped further in the high-dose treatment group when compared to those in control (Table 1B and 2B, protocol #2). Unlike the untreated tumor mice, the muscle loss was severer than the fat loss in the chemotherapeutic agent-treated mice (Table 1A). The final body weight of the normal mice group gained 1% (Figure 1B). On the contrary, the groups receiving 2- or 4-does docetaxel lost 5.5% or 11.6% the body weight, respectively (Figure 1B). After the docetaxel treatment, the food intake declined, suggesting that the body weight loss to some degree is due to anorexia (Table 2B). The levels of serum IL-6 and IL-1β in the group receiving high-dose docetaxel increased, whereas the levels of TNF-α in all groups were more or less the same (Table 2B), suggesting that the docetaxel-induced cachexia differs from the tumor-induced cachexia.

### Chemotherapy inhibits tumor growth but promotes cachexia

**Figure 2:**
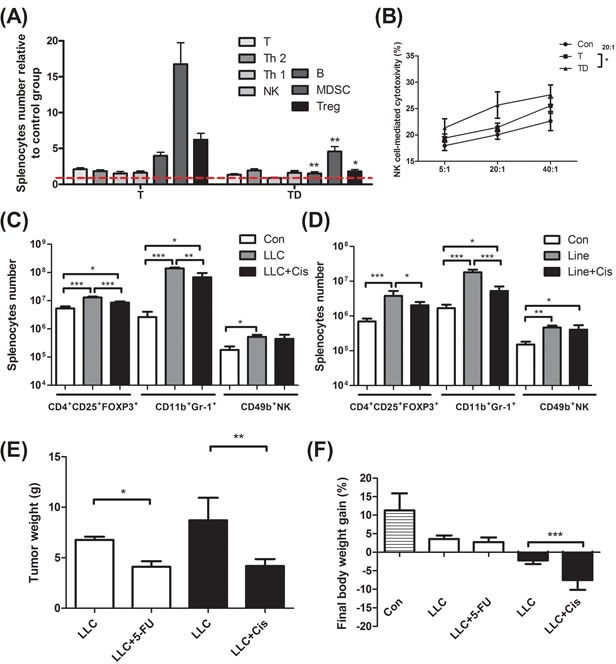
Immunological profiling for mice bearing tumor, mice bearing tumor with chemotherapeutic treatment, and non tumor-bearing mice (Con) (A) Total CD3^+^ T, CD3^+^CD4^+^ T helper 2, CD3^+^CD8^+^ T helper 1, CD49b^+^ NK, CD19^+^ B, Gr-1^+^CD11b^+^ MDSC and CD4^+^CD25^+^Fox-p3^+^ Tregs in the spleen of line-1 tumor-bearing (T) and tumor with docetaxel-treated (TD) mice. The number of splenocyte was normalized with the normal mice (Con) regarded as 1. (B) NK-associated cytotoxicity in the spleen of mice. (C) and (D), Gr-1^+^CD11b^+^ MDSC, CD4^+^CD25^+^Fox-p3^+^ Tregs and CD49b^+^ NK cells in the spleen of tumor-bearing and tumor with cisplatin-treated (Cis) mice. (E and F) After LLC (1×10^5^) tumor implantation, mice were treated with cisplatin (5 mg/kg, every four days) or 5-FU (40 mg/kg, once a week). In the end of experiments, mice were sacrificed and examined for tumor weight (E) and body weight gain (F). Data are shown as mean ± SD. n = 5–9 mice/group and each value is an average of three independent experiments. *p < 0.05, **p < 0.01, and ***p < 0.001 denote levels of significant differences between groups.

In Table 1C, the tumor weight of the docetaxel-treated mice drops about 40%, when compared to that of mice receiving no treatment (Table 1C, protocol #3). It is well known that tumor burden is proportional to the number of splenic immunosuppressive cells [18]. To examine whether the anti-tumor effect of the chemotherapeutic agent is because of attenuation of the tumor-induced immunosuppression, we used ELISA to determine the cell types of splenocytes in mice. Our examination showed that docetaxel markedly reduced the numbers of B, MDSC and Treg cells in tumor-bearing mice, whereas the numbers of T and NK cells were unchanged (Figure 2A). In order to know whether the effect is model-dependent, two new models were examined, where mice bearing Lewis lung carcinoma or line-1 cells were subjected to the cisplatin treatment. As shown in Figure 2C and D, the numbers of MDSC and Tregs cells decline in all cell lines treated with cisplatin. However, the number of NK cells holds unchanged in models treated with docetaxel. It is known that immunosuppressive cells suppress antitumorigenesis of NK cells [28]. To understand if the reduction of immunosuppressive cells enhances the cytotoxicity of NK cells, the NK cells isolated from the tumor-bearing mice were examined. An E/T ratio of 20/1 in the TD group maximized the efficacy on the NK cytotoxicity test (*p < 0.05, Figure 2B) when compared to that in the T group, where only were 25% of line-1 cells stained with PI. Our results suggest that the cytotoxicity of NK cells is compromised in the tumor-bearing mice, whereas the activity can be restored through the chemotherapeutic agent-mediated reduction of immunosuppressive cells.

Chemotherapeutic mice remained suffering weight and muscle losses (Figure 1C, Table 1C, 22.8% loss in T and 47.9% in TD compared with Con group), indicating that although docetaxel reduces tumor burden it indeed assists development of side effects. Consistently, both 5-FU and cisplatin treatments had the similar effect (Figure 2E and F). As shown in Table 2C, docetaxel arouses anorexia, while there is no difference in food intake among testing groups. We reasoned that this is likely due to the docetaxel administration protocol (a four-day interval injection), as we wanted to minimize the adverse cytotoxicity of docetaxel. The levels of serum IL-6 and IL-1β in tumor bearing mice slightly increased after the docetaxel treatment, suggesting that docetaxel exacerbates these side effects.

### TNF-α and myostatin are associated with tumor-induced skeletal muscle atrophy

**Figure 3:**
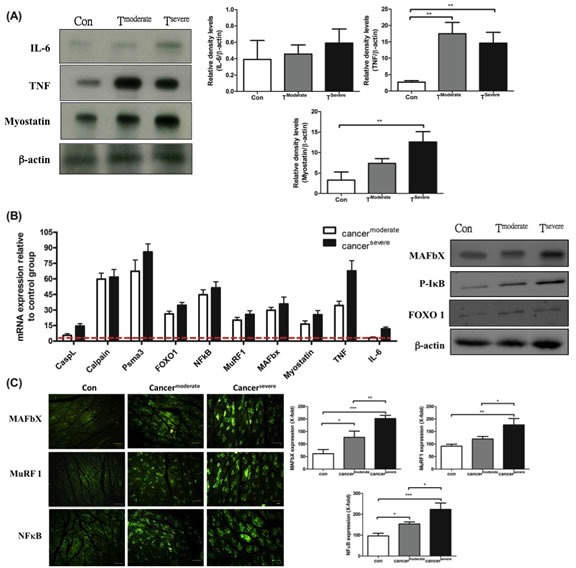
mRNA and protein levels for genes encoding cachexic factors towards proteolytic signaling molecules after line-1 tumor inoculation (protocol #1) (A) Western blot analysis for expressions of IL-6, TNF-α, myostatin and β-actin in gastrocnemius muscles from line-1 tumor-bearing mice. The graph represents relative densitometric intensity of each band normalized to β-actin. (B) mRNA levels (left) and protein levels (right) for genes of cachexic factors and proteolysis relative signaling molecules in gastrocnemius muscle. Values are means of fluorescence signals expressed as a percentage of healthy control mice, and normalization to the GAPDH mRNA amount. (C) Immunohistochemistry of gastrocnemius muscle from tumor-bearing mice, where protein expressions are shown for MAFbx (top), MuRF-1(middle) and NF-κB (bottom). Data are shown as mean ± SD. n = 5–8 mice/group and each value is an average of three independent experiments. *p < 0.05, **p < 0.01, and ***p < 0.001 denote levels of significant differences between groups.

The factors that cause muscle atrophy were examined, we analyzed cytokines and atrophy-related genes in tumor-bearing mice. First, western blotting analysis revealed that: TNF-α was up-regulated in moderate cachexia mice (Figure 3A and B); the serum level of IL-6 was significantly elevated, while its expression in the gastrocnemius muscle remained within the normal range; the serum level of myostatin in the gastrocnemius muscle also increased. Added together, TNF-α and myostatin are likely the key mediators that promote proteolysis.

Several signaling pathways have been implicated in skeletal muscle degradation. To ascertain whether TNF-α and myostatin intrinsically regulate proteolysis, the expression levels of key downstream genes, including *cathepsin* L (a lysosomal protease), *calpain* (autolysis) and *psma*3 (a proteasomal protein), were measured by real-time PCR. As shown in Figure 3B, the mRNAs of psma3, ubiquitin-ligases MuRF-1 and MAFbX in the gastrocnemius muscle of the tumor-bearing mice are up-regulated, suggesting TNF-α is associated with NF-κB signaling pathway through TNF receptors [29]. This reasoning is supported by the fact that up-regulation of NF-κB is proportional to the increase of TNF-α (Figure 3B). One should note that the activation of NF-κB is chiefly via IκB kinase (IKK)-mediated phosphorylation [30].

It has been known that myostatin up-regulates the genes involved in the ubiquitin-mediated proteolysis through the NF-κB-independent, FoxO1-dependent mechanism [31]. The FoxO-1 transcription factors can induce skeletal muscle wasting via regulating the atrophy-related genes, namely, the ubiquitin ligase MAFbX and Cathepsin-L [32, 33]. The mRNA and protein levels of FoxO-1 markedly increased in both moderate and severe cachectic mice (Figure 3B). Though FoxO-1 is related to lysosomal atrophy, the mRNA level of cathepsin-L only slightly increased in our experiment (Figure 3B). To confirm that the tumor-induced muscle atrophy truly follows the ubiquitin-proteasome pathway, the expression of ubiquitin ligases was examined by indirect immunofluorescence assay. As shown in Figure 3B and C, the protein levels of MAFbX and MuRF-1 increase in the gastrocnemius muscle of the tumor-bearing mice. Taken together, the tumor-induced muscle atrophy is likely due to elevated levels of TNF-α and myostatin, which act together to activate NF-κB and FoxO1 so as to amplify the expressions of calpain (autolysis) and ubiquitin ligases (ubiquitin-proteasome) (Figure 5E).

### IL-6 affects skeletal muscle proteolysis in chemotherapy

**Figure 4:**
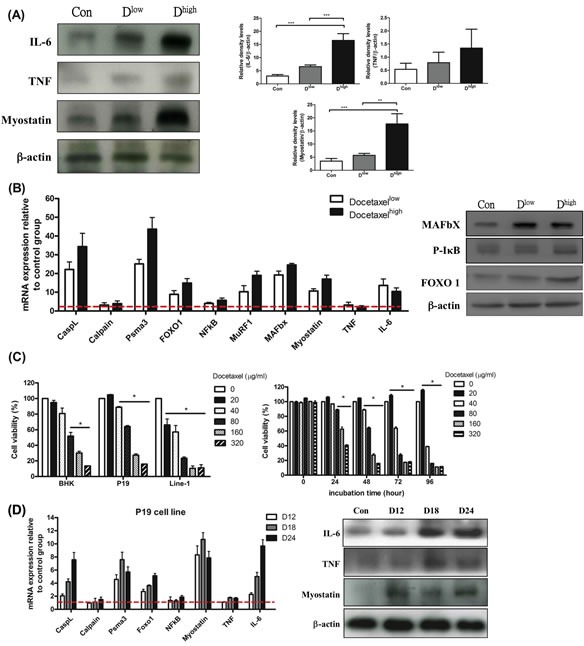
mRNA and protein levels for genes encoding cachexic factors and proteolytic relative signaling molecules in docetaxel-induced muscle atrophy *in vitro* and *in vivo* (protocol #2) (A) Western blot analysis for expressions of IL-6, TNF-α, myostatin and β-actin in gastrocnemius muscle. The graph represents relative densitometric intensity of each band normalized to β-actin. (B) mRNA levels (left) and protein levels (right) for genes encoding cachexic factors and proteolysis relative signaling molecules in gastrocnemius muscle from docetaxel injected mice. (C) Cells at 0.5 × 10^4^ were plated per well, and incubated for 24 hr. The cells were treated with various concentrations of docetaxel for 48 hr and the cell viability was measured by the MTS assay (left). P19 cells were treated with a range of concentrations of docetaxel for 0, 24, 48, 72 or 96 hr, and the cell viability was measured by the MTS assay (right). Data are relative to control. (D) The time course effect of docetaxel (80 μg/ml) on mRNA and protein expression related to protein degradation in P19 muscle cell line over 24 hr. Values are means of fluorescence signals expressed as a percentage of healthy control mice or normal P19 cells, which are normalized to the GAPDH mRNA amount. Data are shown as mean ± SD. n = 5–10 mice/group and each value is an average of three independent experiments. *p < 0.05, **p < 0.01, and ***p < 0.001 denote levels of significant differences between groups.

Agreeing with our previous report, the skeletal muscle wasting of mice in chemotherapy brought about immediately after the onset of cachexia (Table 1B). The effects of docetaxel on skeletal muscle proteolysis in healthy mice were examined as a contrast. Western blotting analysis for the gastrocnemius muscles of normal adult mice receiving a high dose of docetaxel revealed that the levels of both IL-6 and myostatin increased 3 folds relative to that of TNF-α (Figure 4A). To know whether docetaxel affects normal BHK-21 or specific muscle P19 cells, cytotoxicity was examined by MTS assay. Cells were cultured in the presence of 0, 20, 40, 80, 160, 320 μg/ml docetaxel for 48 hr and then subjected to the assay. The result showed that docetaxel inhibited proliferation of line-1 cells in a dose-dependent manner (Figure 4C). The inhibitory level of docetaxel for BHK-21 cells was lower than that for line-1 cells. Namely, docetaxel at a dose of 40 μg/ml can kill 50% of line-1 cells but kill only 10% of BHK-21 or P19 cells (Figure 4C). Interestingly, the P19 skeletal muscle cells can restore its population when exposed to the low dose of docetaxel (Figure 4C). We further performed a time-course assay, where the growth of P19 cells increased 20% in the presence of 20 μg/ml docetaxel (Figure 4C right). The docetaxel-treated mice however showed lethal wasting syndrome, a progressive muscle/weight loss (Table 1B).

**Figure 5:**
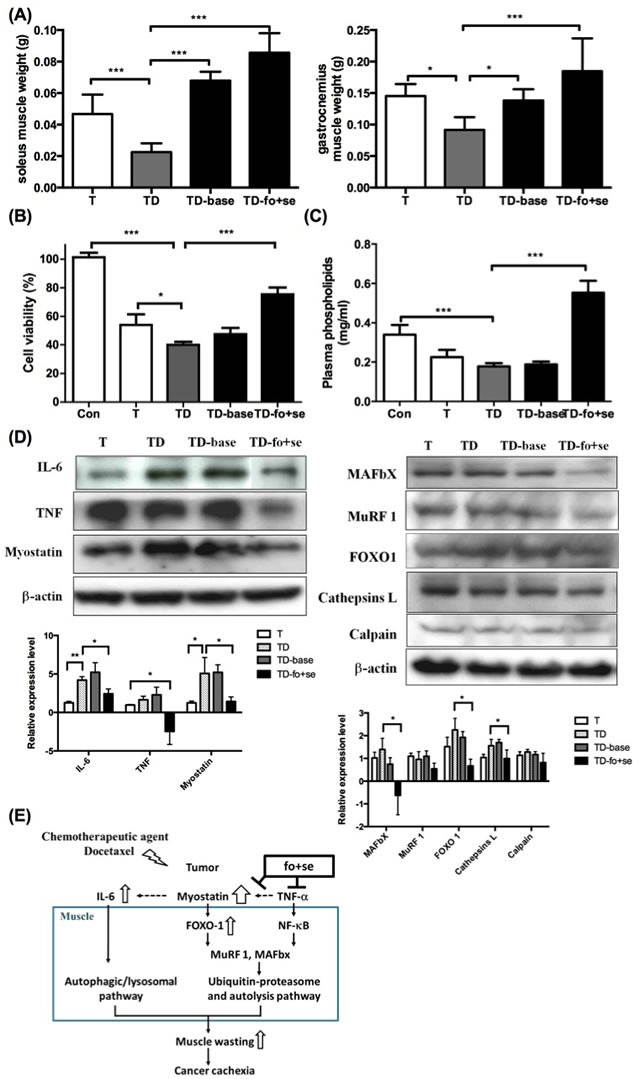
Effects of daily oral administration of background diet or combined nutritional components (addition of fish oil (fo) or selenium yeast (se)) on skeletal muscle atrophy in tumor-bearing mice with chemotherapy (protocol #3) (A) differences in soleus and gastrocnemius weights at day 42 after intervention with nutritional supplements in tumor-bearing mice after chemotherapy. (B) Effects of serum from mice on P19 cell growth. Serum (10% of assay volume) was added to the P19 cell line, and the cell viability during 24 hours incubation was determined by the MTS assay. (C) Concentrations of plasma phospholipids [16] fatty acids in mice. (D) Western blots analysis for expressions of IL-6, TNF-α and myostatin in gastrocnemius muscles of mice (left). Protein levels for ubiquitin ligases, FoxO-1, cathepsin L and calpain in gastrocnemius muscle during cancer and chemotherapy (right). (E) The model illustration for fish oil plus selenium attenuated muscle atrophy after chemotherapy. Under chemotherapy, tumor-bearing mice exhibited a significant increase in the expression of myostatin that activates FoxO-1, and leads to up-regulation of proteasome ubiquitin ligases MuRF-1 and MAFbX. Con, normal control mice; TD, tumor-bearing mice receiving docetaxel; TD-base, tumor-bearing mice receiving docetaxel and background diet; TD-fo+se, tumor bearing mice receiving background diet with additional fish oil and selenium yeast. Data are shown as mean ± SD. n = 5–9 mice/group and each value is an average of three independent experiments. *p < 0.05, **p < 0.01, and ***p < 0.001 denote levels of significant differences between groups.

It is now clear that myostatin provokes muscle atrophy through an NF-κB-independent, FoxO1-dependent mechanism [16] and that IL-6 mediates muscle protein degradation by enhancing lysosomal cathepsin proteolysis [34]. Our assays showed that FoxO-1 was up-regulated and that NF-κB was kept steady in the gastrocnemius muscle of the docetaxel-treated mice (Figure 4B). The real-time PCR analysis also demonstrated that the expressions of ubiquitin ligases and cathepsin-L increased in the gastrocnemius muscles of normal adult mice (Figure 4B). The reporter analysis further confirmed that both lysosomal cathepsin and ubiquitin-proteasome increased with addition of docetaxel to P19 muscle cells in a time-dependent manner (Figure 4D). Interestingly, when P19 muscle cells were treated with docetaxel for 12 hr, the myostatin mRNA or its protein expression increased, whereas IL-6 or TNF-α was the same (Figure 4D). Added together, the docetaxel-induced muscle atrophy is likely following the ubiquitin-proteasome and lysosomal pathways through the myostatin-mediated FoxO1-dependent mechanism; the resulting IL-6 then up-regulates atrophy-related genes (Figure 5E).

### Combination of fish oil and selenium attenuates chemotherapeutic induced muscle proteolysis through inhibiting FoxO-1 signaling

Recent clinical studies revealed that skeletal muscle depletion is related to reduction of plasma n-3 fatty acids in NSCLC patients [21]. For example, Fearon et al. [35] reported that there is a positive correlation between plasma EPA and lean body mass in advanced cancer patients receiving fish oil supplementation. Similarly, our study suggested that tumor-bearing mice supplemented with a combination of fish oil and selenium yeast significantly increased their body weights [18]. Since few therapeutic options are available to cope with cancer cachexia, nutritional supplementation appears to be a promising measure to sustain muscle masses against cancer cachexia.

As shown in Figure 5A, the weights of soleus and gastrocnemius muscles lose significantly in tumor-bearing mice under chemotherapy. This situation was reversed by supplementing mice with nutrients (TD-fo+se). One should note that background diet slightly improved muscle atrophy, likely because the diet contained some essential nutrients, for example, branched chain amino-acid (BCAA) leucine [36]. Since the level of phospholipids (PLs) is a metabolic index of endogenous or dietary fatty acids [37], the level of plasma PLs was therefore determined to estimate n-3 fatty acids in the blood of mice. As shown in Figure 5C, the PLs level in tumor-bearing mice under chemotherapy (TD) is lower than that of normal mice [26]. In contrast, the level of PLs in mice supplemented with fish oil and selenium yeast is higher.

Growing evidence suggests that the tumor inflammatory responses are positively correlated to progression of cachexia. Key inflammatory factors IL-1β, IL-6 and TNF-α in serum were thus determined. The IL-6 level significantly increased in tumor-bearing mice after chemotherapy (Table 2C), suggesting that both chemotherapeutic agent-induced IL-6 and myostatin promote muscle atrophy. We further examined the proliferation rate of P19 muscle cells treated with sera from different groups of mice, where the viability of the cells treated with serum from mice receiving no supplementation significantly decreased (Figure 5B). In contract, the viability of the cells treated with the serum of TD-fo+se or TD mice increased to 78% or 40% (Figure 5B), underlining the effect of fish oil and selenium.

To probe which mediators are involved in the regulation of the chemotherapeutics or nutritional supplementation-mediated proteolysis, the expression of proteolytic-related proteins in gastrocnemius muscles of cachectic mice was examined. As shown in Figure 5D, myostatin and IL-6 significantly increase in the docetaxel-treated mice (TD), as opposed to that in tumor-bearing mice (T). The myostatin-mediated FoxO-1 was also up-regulated after chemotherapy (Figure 5D right). Our results suggest that both docetaxel-elicited myostatin and IL-6 up-regulate FoxO-1 signaling pathway, thus facilitating muscle degradation. Administration of fish oil and selenium inhibits expression of cytokines and myostatin in cachectic mice, thus down-regulating the proteolytic signaling pathway (Figure 5E).

## DISCUSSION

Cachexia, a debilitating syndrome, occurs in many cancers, while the causation remain poorly understood. Cachexia is generally associated with systemic inflammation, enhanced proteolysis, and hypoalbuminemia. Identifying committed biomarkers may provide a solution to cope with cancer cachexia. It is also known that antineoplastic therapies such as chemotherapy influences systemic inflammation and wasting, the mechanism of which is again less elucidated.

In this study tumors that cause significant decline of mice body weight and serum albumin may account for the fact that human cancer wreaks havoc on man (Figure 1, Table 2). Cancer cachectic mice showed losses of muscle and fat; the latter was more seriously affected at the early onset of the disease (Table 1A). The body composition in chemotherapeutic agent-induced cachexia differed from that receiving no therapy, namely, muscle loss is quicker than fat loss in the former (Table 1B), while the trend reverses in the later. The mice with tumor-induced cachexia may experience more severe loss of skeletal muscles; mice receiving a higher dose of the chemotherapeutic agent likely developed anorexia. So, the energy utilization in brain may switch from glucose to fat-derived ketone bodies thereby slowing down gluconeogenesis from amino acids in liver.

The docetaxel treatment deteriorated muscle wasting in tumor mice (Table 1C), suggesting that the agent plays a dual role for muscle atrophy and tumor shrinking (Figure 2). Gemcitabine, a nucleoside analog, is used to reduce the number of pro-inflammatory MDSC thus improving anti-tumor responses [38]. Docetaxel significantly reduced the number of MDSC and Tregs in spleen of tumor-bearing mice, while the numbers of CD4^+^ T cells, CD8^+^ T cells and NK cells were not changed (Figure 2A). Since TNF-α promotes expansion and function of Tregs through TNF receptor type II (TNFRII) [39], the reduction of Tregs may be ascribed to the decrease of TNF-α upon docetaxel administration. Inflammatory cytokines TNF-α and IL-6 are two key mediators in skeletal muscle homeostasis [40]. In this study the circulating level of IL-6 was constant from one group to another, suggesting that TNF-α is more critical than IL-6 in tumor-induced muscle wasting (Table 2A).

Protein degradation is regulated by three major proteolytic pathways, ubiquitin-proteasome pathway, calcium-activated system and lysosomal pathway. Recent studies suggested that TNF-α-dependent NF-κB initiates both the ubiquitin-proteasome pathway [41] and the calcium-activated system in protein degradation [42]. Myostatin, a member of the TGF-β superfamily of secreted growth factors, negatively regulates skeletal muscles (Figure 3) [43]. The fact is that myostatin down-regulates the expression of myogenic genes MyoD and pax3, in contrast to the ubiquitin- and lysosomal-associated genes that are up-regulated [44]. Myostatin also inhibits phosphorylation of Akt, thus increasing FoxO-1 but not NF-κB [31]. As a result, TNF-α and myostatin together up-regulate both ubiquitin-proteasome and calpain systems for disassembly/degradation of muscle myofilaments (Figure 5E).

In this study healthy mice injected with docetaxel showed acute skeletal muscle wasting and elevation of IL-6 in serum and muscle, suggesting that IL-6 is the key factor in the induction of cachexia (Table 2B). The level of myostatin increased earlier than that of IL-6 in P19 muscle cells treated with docetaxel (Figure 4D), suggesting that myostatin interacts with circulating inflammatory cytokines. This result agrees with the study where C2C12 myotubules treated with myostatin significantly increased IL-6 mRNA in muscle cells [45], while it disagrees with some published results that favor the proteasome-dependent muscle proteolysis [26, 46]. In general, skeletal muscle atrophy results from overexpression of TNF-α and myostatin, which up-regulates calcium-activated and ubiquitin-proteasome systems in tumor mice. In contrast, docetaxel increases myostatin to activate FoxO-1, thus leading to more serious protein degradation.

Our result demonstrated that the muscle in the line-1 tumor-bearing mice treated with docetaxel underwent atrophy 1.5 folds severer than that treated with tumor cells alone (Table 1C), indicating that the chemotherapeutic agent causes muscle atrophy and tumor shrinking. One should note that the muscle mass increased but the expression of proteolytic-related proteins had no change in the TD-base group. Our results suggest that BCAAs (leucine in particular) play a role in the regulation of skeletal muscle protein metabolism [47]. The background diet that contains BCAAs (isoleucine, leucine and valine) probably has no role on proteolysis, but instead it may stimulate protein synthesis.

Growing evidence has shown that the cancer-provoked inflammatory responses promote serious cachexia. Imbalanced catabolism/anabolism is evident not able to maintain lean body mass even under normal food intake [48]. Clinical data further [49] suggested that n-3 fish oil can reduce both catabolism and body weight loss in cancer patients. The increased survival rate for advanced cancer patients supplemented with n-3 fatty acids was ascribed to reduction of inflammatory responses [50]. On the other hand, Vieira et al. (2015) reported that supplementation with selenium can reduce chemotherapeutic side effects for cancer patients [17]. It has been suggested that selenium is a modulator in circadian clocks, that protects mice from toxicity of a given chemotherapeutic drug [51].

In previous study we demonstrated that dietary supplementation with fish oil and selenium synergistically reduced immunosuppressive cells in tumor-bearing mice [18]. The additive effect of these two nutrients is best illustrated by the change of skeletal muscle masses shown in Figure 5. This additive effect can be explained by two facts: [1] anabolic stimulation by essential amino acids, [2] reduction of protein catabolism by reduced inflammatory (fish oil and selenium) and down-regulation of proteolysis signaling pathway. With supplementation of both fish oil and selenium the skeletal muscle atrophy improved significantly is likely due to down-regulation of myostatin as well as related cytokines. Fish oil may balance body compositions in cachectic patients particularly by reducing inflammatory responses. Our result echoes a recent study, where eicosapentaenoic acid (EPA) eased adverse effects of TNF-α on C2C12 myogenesis [52]. Similarly, supplementation with EPA and DHA for advanced cancer was able to lessen lean tissue wasting [35]. Moreover, our results showed that reduction of both TNF-α and myostatin only occurred in the TD-fo+se group but not in the TD-base group. The reduction of myostatin in muscle is likely through inhibiting humoral mediator(s) secreted by the implanted tumor cells or host cells in response to chemotherapy. As a result, fish oil and selenium yeast can antagonize loss of skeletal muscle proteins in cancer cachexia by down-regulating proteolytic genes expression; this may also be the mechanism whereby tumor growth is inhibited. As to how individual fish oil or selenium takes part in the proteolytic protection, the effect requires further examination.

Elevation of serum IL-6 and IL-1β in tumor-bearing mice after docetaxel treatment implicates that docetaxel gives rise to side effects. To know that this is not specific to docetaxel, we also examined 5-FU and cisplatin, which reduced not only the tumor burden but also the body weight. Although protein synthesis was affected, skeletal muscle protein degradation is likely the major cause for cachexia [53]. In addition to the reduction of immunosuppressive cells [18], the combination of fish oil and selenium can restore the muscle mass by inhibiting the proteolytic pathway.

In summary, specific immunocompetent lung cancer mice models established in this study demonstrated that cancer cachexia is closely related to chemotherapy. The anti-cancer drug docetaxel modulated immune responses in suppression of Tregs and MDSC, thus aggravating muscle wasting. The model system developed here is suitable to serve as a research platform, for example, for probing muscle atrophy mechanisms/lung cancer immune regulation as well as evaluating therapeutic efficacy for new drug candidates. Importantly, the combination of the selected nutrients (fish oils and selenium yeast) not only attenuates muscle protein degradation but also stimulates protein synthesis in chemotherapy. Despite requiring further clinical evaluation, this supplement regimen in conjunction with conventional cancer therapy bodes well for future cancer therapy.

## METHODS

### Cells and cell culture

Line-1 cells from a BALB/cByJ alveolar lung carcinoma (provided from Dr. John Yuhas) were adapted to tissue culture [54]. Line-1 and baby hamster kidney-21 cells (BHK-21, ATCC CCL10) were maintained in RPMI-1640 medium supplemented with 5% and 10% fetal bovine serum, respectively. Lewis lung cancer cells (LLC, CRL-1642) were maintained in Dulbecco's modified Eagle's medium. Mouse embryonic teratocarcinoma cells (P19; ATCC CRL-1825) were differentiated in the presence of 0.5% dimethylsulfoxide (DMSO) to form cardiac and skeletal muscle-like elements [55]. Undifferentiated cells were propagated in DMEM (GIBCO-BRL Burlington, ON, Canada) supplemented with 10% heat-inactivated FBS, and the antibiotics (GIBCO/BRL) penicillin G (50 units/ml) and streptomycin (50 cg/ml). The cultures were maintained at 37°C in a humidified atmosphere of 5% CO_2_ and passaged every 2 days. Differentiation was routinely induced with DMSO. Briefly, 2.5 × 10^6^ cells were allowed to aggregate for 4 days in nonadhesive bacteriological grade Petri dishes (6 cm diameter) containing 5 ml of complete medium, in the presence of 0.5% DMSO (Sigma). Cardiac muscle first appears on Day 6 of differentiation and skeletal muscle on Day 9.

### P19 cell treatments and extract preparation

P19 cells were cultured as described in 4.2.1. After 9 days incubation with 0.5% DMSO, cells were cultured with DMEM (contain 0.5% DMSO) in the presence or absence of 80 μg/ml docetaxel. After 12, 18 or 24 hr, medium was removed and collected into eppendorf. Cells and then washed in PBS and lysis buffer (25 mM Tris-HCl, pH 7.4, 0.5 mM EGTA, 25 mM NaCl, 1% Nonidet P-40, 10 mM NaF, 0.01 mg/ml leupeptin, 1 mM orthovanadate, 100 nM okadaic acid, 1 mM benzamidine, 2 mM AEBSF) was then added to the cells, and the lysate was centrifuged at 12,000 rpm for 10 min. All samples were stroed at −80°C until further analysis.

### Cytotoxicy assay

Cell viability was monitored by using the CellTiter 96 Aqueous One Solution Assay and performed according to the manufacturer's instructions (Promega Corporation, USA). In brief, LLC, Line 1, BHK-21 and P19 cells at a desity of 0.5 × 10^4^ in 80 μl/well were incubated in a 96-well plate at 37^°^C in a humidified 5% CO_2_ atmosphere. After 24 hours cells were then treated with different concentration of docetaxel (320, 160, 80, 40, 20 μg/ml) for 48 hours. On the other hand, P19 cells were incubated in DMEM in the presence of 10% serum from mice for 24 hours. After that MTS solution was added (20 μl/well) and cells were incubated for 2 hours under the same conditions. At the end of incubation, the absorbance at 490 nm was measured using a BIOTEK μQuant microplate reader (BIO-TEK instrument, Winooski, VT).

### Animals, tumor implantation, and chemotherapy

Male BALB/cByJ or C57BL/6 mice (6-7 weeks) were obtained from the National Laboratory Animal Center. Mice were individually housed in a climate controlled room (12:12 dark-light cycle with a constant room temperature of 21±1^°^C). Mice were given at least 4 days to adjust to their new environment and diet before treatments were imposed. Mice were given free access to water and food (laboratory rodent diet, labdiet 5001, USA). After acclimatization mice were divided into weight-matched groups. In the first tumor model (Figures S1, protocol #1), mice were inoculated s.c. with a homogenate of tumor cells (1×10^5^) on day 0. The control group was injected with 0.1 ml of sterile saline solution. In the second chemotherapeutic model (Figures S1, protocol #2), animals received two or four times i.p. injection of either docetaxel (taxotere®, 20 mg/kg body weight) or saline every day to create an acute response. The third tumor combine chemotherapeutic model (Figures S1, protocol #3), after tumor implantation, the mice were treated with docetaxel (20 mg/kg) or cisplatin (5 mg/kg) every four days, or 5-FU (40 mg/kg) to approach clinical observation. Following inoculation of tumor cells or drug, body mass, food intake and tumor size were measured four times a week. Tumor growth was assessed by the measurement of two bisecting diameters in each tumor using calipers. The size of the tumor was determined by direct measurement of the tumor dimensions.

### Experimental diets

The mice in the dietary supplementation experiment were daily supplemented with 63 mg fats (100% soy oil), 514 mg carbohydrates and 194 mg proteins as a background diet (Ethan Nutraceutical Company Ltd., USA). The experimental diets included additional 20 mg fish oil (contained 11 mg EPA and DHA) and/or 0.69 μg selenium yeast. Fish oil and selenium yeast were obtained from Ethan Nutraceutical Company Ltd. (USA).

### Levels of serum albumin, cytokines and phospholipids levels

The levels of serum albumin were measured for experimental mice using the SPOTCHEM EZ SP-4430 dry chemical system (Arkray, Kyoto). Cytokines in serum were measured using the OptEIA™ ELISA Set (BD, Canada, USA) for mouse TNF-α, IL-6 and IL-1β. Optical density was recorded using a μQuant spectrophotometer (Bio-Tek Instruments, Winooski, USA). Plasma phospholipids [16] levels were measured in experimental mice by using a SPOTCHEM EZ SP-4430 dry chemical system (Arkray, Kyoto).

### Splenocyte isolation and flow-cytometric analysis

Splenocytes were isolated by centrifugation (300 g); red blood cells were lysed using the Gey's reagent (0.829 g NH4Cl, 0.1 g HCO_3_ and 3.72 mg Na_2_EDTA in 100 ml ddH_2_O). For determining phenotypes of splenocytes isolated from spleens, cells were stained with an appropriate combination of anti-CD3ε (100306; Biolegend), anti-CD4 (100516; Biolegend), anti-CD8a (100714; Biolegend), anti-CD19 (115508; Biolegend), anti-Gr-1 (108416; Biolegend), anti-Ly6G (127613; Biolegend) or anti-CD11b (101212; Biolegend) after blocking of the Fc receptor with anti-CD32/CD16 (BD Biosciences) at 4^°^C. For T regulatory cells staining, cells were incubated with anti-CD4 and anti-CD25 (102006; Biolegend) for 30 minutes, followed by fixing in 2% paraformaldehyde, permeabilizing with Perm/Wash buffer (BD Biosciences), and staining with anti-Fox-p3 (320008; Biolegend). For determining NK cytotoxicity, cells were isolated from mouse spleens (regard as effector cells). The target cells (line-1) were stained with DIOC18 (10 μl per 1×10^6^ cells) for 20 min at 37°C. The cells then were washed twice with a buffer solution and then resuspended in a complete culture media at a concentration of 1×10^6^ cells/ml. The target and effector cells (splenocytes) were prepared by making co-cultured cells in ratios of E:T= 5:1, 20:1, and 40:1. These co-cultures were incubated for 4 hours, and centrifuged at 250 g for 5 min through alternate washes and suspensions; supernatants were discarded. The cells then were labeled with propidium iodide (PI, 2 μl/per test) and incubated at room temperature in dark. Analysis was performed using FACScan (BD Biosciences). For characterization of cell types, a large gate was set to include monocytes and lymphocytes for forward scatter vs. side scatter.

### RNA extraction and RT-qPCR

Total tumor RNA was extracted with a commercially available RNA mini kit (Qiagen); cDNA was synthesized using the M-MLV reverse transcriptase (Promega) and the oligo-dT15-primer (Promega). Real-time qPCR primers were designed using the Primer3 webware; electrophoresis was performed to verify DNA products. Reactions were run on the Bio-Rad iCycler iQ system in the presence of the Sybr-Green PCR mix (iCycler iQ Real Time PCR Detection System, Bio-Rad). The comparative threshold cycle [54] method was used to calculate the relative expressions [56]. For quantification of gene expressions, the values of target genes were normalized by the value of the endogenous reference GAPDH. The quantity of the target gene relative to a calibrator (normal pool expression) is given by: 2^−ΔΔC^_T_ [ΔC_T_ = C_T_(target gene) − C_T_(GAPDH); ΔΔC_T_ = C_T_ for any sample − ΔC_T_ for the calibrator].

### Protein extraction and western blotting

Proteins from tumors were extracted with a buffer solution (20 mM Tris-HCl, pH 7.5, 2 mM ATP, 5 mM MgCl_2_, 1 mM dithiothreitol (DTT), and 5 μL of a protease inhibitor cocktail (Sigma)). Proteins (20 μg/lane) were separated on a 12.5% polyacrylamide gel (a precast SDS gel (Bio-Rad)) and then transferred to a polyvinylidene difluoride membrane (Immobilon, Millipore). Proteins were determined using antibodies against mouse IL-6 (1:200, Abcam), TNF (1:200, Santa Cruz Biotechnology), Myostatin (1:100, Abcam), MAFbX (1:200, Santa Cruz Biotechnology), MuRF 1 (1:100, Novus), Cathepsina L (1:100, Abcam), Calpain (1:100, Santa Cruz Biotechnology), p-IκB-α (1:100, Santa Cruz Biotechnology) and FOXO1 (1:200, Abcam). The antibodies then were stripped off the membrane and re-probed with a specific antibody against β-actin (1:5000, Novus Biologicals). The intensity was quantified using the Fotodyne Image analysis System (Fotodyne, Hartland, WI, USA) and the TotalLab software (Nonlinear Dynamics, Durham, NC, USA).

### Immunofluorescence assay

Paraffin sections were blocked by a blocking buffer for 1 hour at room temperature and stained with a specific primary antibody at a dilution of 1:200 for 24 hours. The primary antibody was washed using PBS. The sections then were stained with a specific secondary antibody at a dilution of 1:100 for 24 hours at room temperature and washed with PBS. The primary antibodies used here are listed as follows: rabbit anti-mouse NF-κB (Novus, USA), rabbit anti-mouse MuRF-1 (Santa Cruz, USA) and FITC-conjugates rabbit anti-mouse MAFbx (ECM biosciences, USA). The secondary antibody was the FITC-conjugates goat anti-rabbit IgG (Sigma, USA).

### Statistical analysis

Data were expressed as means ± SD. Statistical significance was determined by one-way ANOVA followed by Bonferroni's multiple comparison test (Prism Graph Pad). Differences were considered statistically significant when P<0.05.

## SUPPLEMENTARY MATERIAL


