# Supplementary material for: Skeletal muscle atrophy is attenuated in tumor-bearing mice under chemotherapy by treatment with fish oil and selenium
Source: Oncotarget. 2015 Mar 8;6(10):7758–73. doi: 10.18632/oncotarget.3483 (PMC4480714; doi:10.18632/oncotarget.3483)
Supplement: Supplementary file 1 [file oncotarget-06-7758-s001.pdf]

Skeletal muscle atrophy is attenuated in tumor-bearing mice under chemotherapy by treatment with fish oil and selenium

Supplementary Material

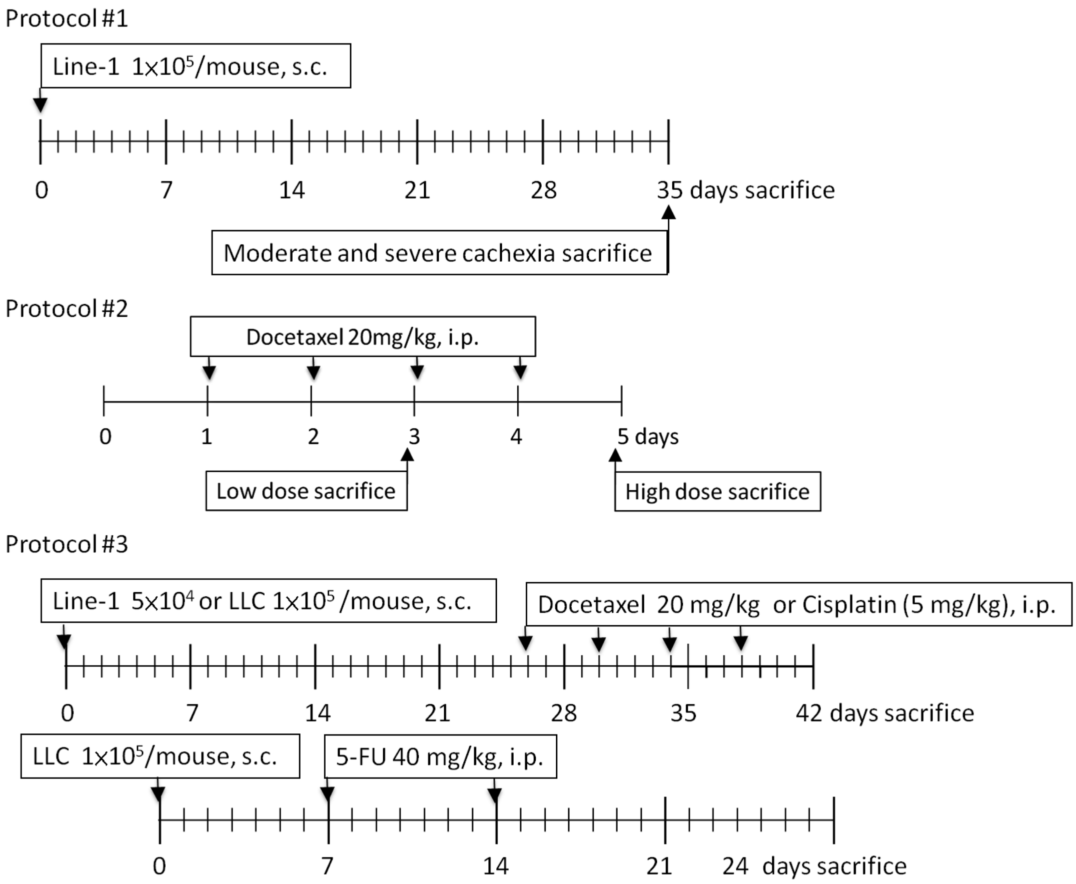

Figure S1. Protocols for tumor- and/or chemotherapeutic agent-induced cachexia in mice. In protocol #1, BALB/cByJ mice were inoculated subcutaneously (s.c.) with a homogenate of line-1 tumor cells ($1x10^5$) on day 0. The control group was injected with 0.1 ml of sterile saline solution. In the protocol #2, BALB/cByJ mice received two or four i.p. injections of either docetaxel (taxotere®, 20 mg/kg body weight) or saline every day. In protocol #3, after tumor implantation the mice (BALB/cByJ or C57BL/6) were treated with docetaxel (20 mg/kg) or Cispatin (5 mg/kg) or 5-FU (40 mg/kg) by i.p. injection.
